# Analytic framework for understanding the competing multiple light scattering processes

**DOI:** 10.1038/s41598-019-39165-7

**Published:** 2019-02-26

**Authors:** Ye-Ryoung Lee, Wonjun Choi, Seungwon Jeong, Wonshik Choi

**Affiliations:** 1 Center for Molecular Spectroscopy and Dynamics, Institute for Basic Science, Seoul, 02841 Korea; 2 Department of Physics, Korea University, Seoul, 02841 Korea

## Abstract

In many complex physical phenomena such as wave propagation in scattering media, the process of interest often cannot be easily distinguished from other processes because only the total combined process is accessible. This makes it difficult to extract the precise knowledge of each subprocess. Here, we derive an analytic expression describing the way the eigenchannel coupling of the total process distributes its energy to the individual subprocesses, with only partial information on each subprocess such as the average eigenvalue 〈*τ*〉 and enhancement factor *η*. We found that the ratio of (*η* − 1)〈*τ*〉 between two subprocesses is a critical parameter determining the preferable subprocess in the energy coupling. This work provides a new analytic framework for understanding the effect of wavefront shaping in the control of wave propagation in disordered media.

## Introduction

The control of waves propagating through complex media has attracted significant attention due to its potential practicality and underlying physics. For example, delivering acoustic or optical waves to target objects embedded within inhomogeneous biological tissues has been a necessity for enhancing image contrast, disease treatments, and stimulating biological functions. Similarly, it is critical that the energy of microwaves is focused through reverberant scattering environments to target antennas in order to maintain the optimal efficiency of the information transfer. Previously, controlling the waves inside complex media was considered a difficult task as the waves become diffused by random multiple scattering. However, the technical advance in wavefront recording/shaping devices, and the use of intriguing physics of wave propagation, mainly time reversal, memory effect, and long-range wave correlation^1,2^, have made it possible to deterministically compensate wave distortion.

In the field of optics, the phase conjugation of monochromatic waves was implemented by using either analog holography^3^ or nonlinear crystal^4^ to reverse the image distortion by a scattering medium. With the advance of the liquid-crystal digital spatial light modulator (SLM), focusing of an optical wave to a spot behind a scattering layer was demonstrated by the iterative feedback control of SLM^5–8^. A generalized approach of using the transfer matrix of the scattering medium was proposed for image delivery and focusing through a scattering medium^9,10^. In acoustics and microwaves, the iterative time reversal operation, an equivalence of the phase conjugation operator, was implemented to refocus distorted waves by the scattering media back to its original source^11,12^. The combination of acoustics and optics was also reported, where optical phase conjugation was applied to refocus acoustically modulated optical waves back to acoustic focus^13–16^. In addition to the undoing of the wave distortion, it was demonstrated that wave energy transmission through scattering media can be enhanced by exploiting the long-range wave correlation induced by a scattering medium^17–21^. However, most of these studies remain remote from *in vivo* applications because the site where waves are controlled is located outside of the scattering medium.

For more realistic applications, efforts have been made to focus waves to the target objects embedded within scattering media. In the case when highly reflecting scattering particles are embedded within a weakly scattering medium, it was shown that the iterative time reversal operation leads to the focusing of waves to the most reflecting particle^22^. A more generalized approach was to couple waves to the individual eigenchannels of the monochromatic transfer matrix measured in the backscattering geometry in acoustics^23^ and optics^24^. By using the formalism decomposing the transfer matrix into a propagation matrix from the input plane to the scatterer’s plane and the diagonal reflectance matrix describing the target scatterers, it was shown that individual eigenchannels are associated with the waves focused on the individual scatterers with distinct reflectivity. However, this decomposition cannot be applied to the case of a highly scattered medium since such multiple-scattered waves that have no interaction with target objects dominate the backscattered waves reflected by the scatterers in the measured monochromatic transfer matrix.

**Figure 1:**
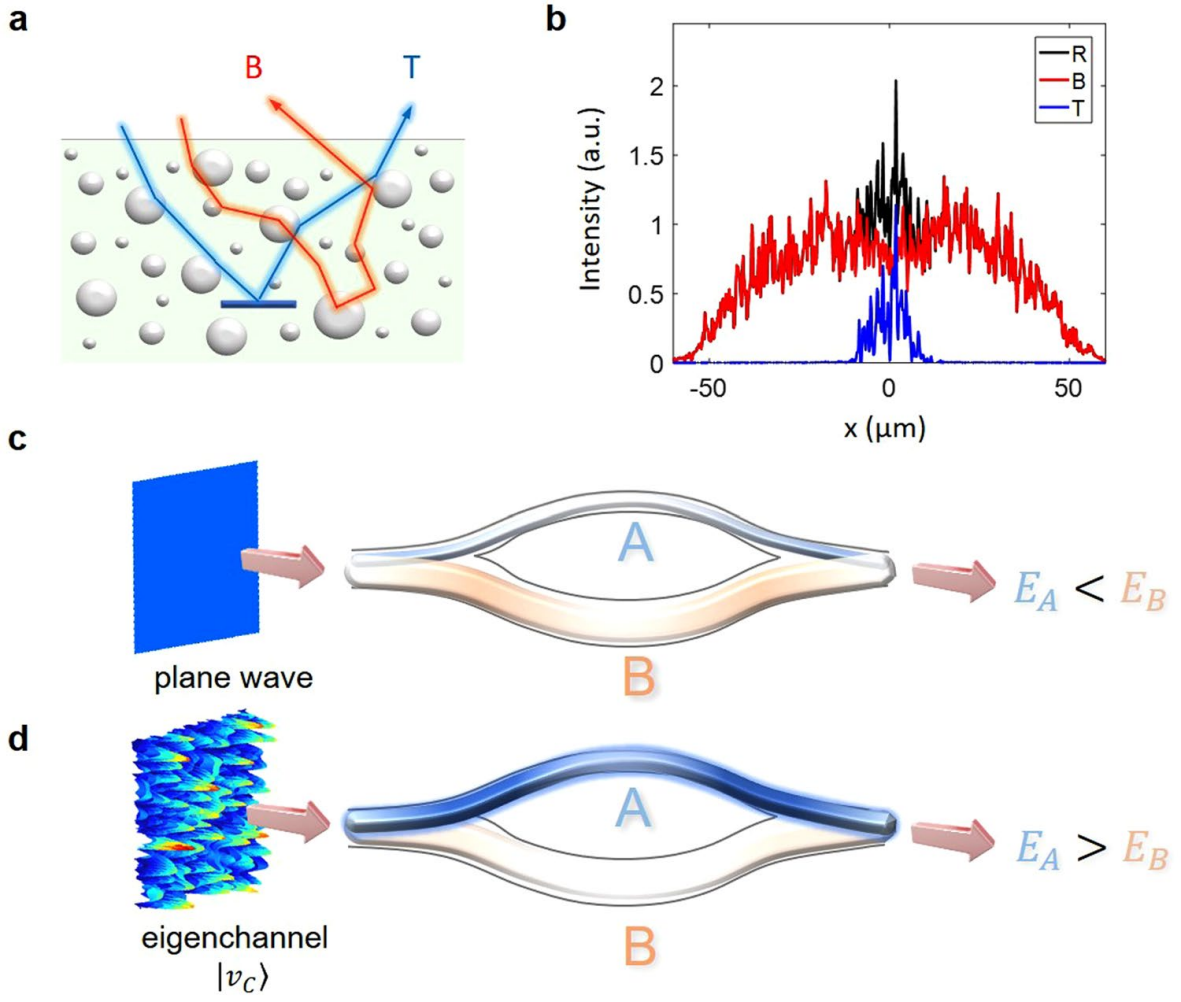
Competing light scattering processes. (**a**) Schematic trajectories of wave propagation for a scattering medium with an embedded target. Multiple-scattered waves that have not interacted with target (*B*) and those that have interacted with target (*T*) are shown in red and blue, respectively. (**b**) Reflection intensity images with (*R*, black) and without (*B*, red) the target. This result was obtained by the FDTD simulation for the sample considered in Fig. 4. The image only from the target (*T*, blue) can be extracted from the difference between *R* and *B*. (**c**,**d**) Two linear transformation operators, *A* and *B*, form a merged operator, *C*. Input and output ends are fused such that *A* and *B* are inseparable. For example, the energy *E*_*A*_ delivered through A is smaller than *E*_*B*_ when an uncontrolled input is coupled (**c**), but *E*_*A*_ can be larger than *E*_*B*_ when the eigenchannel of the merged operator *C* (|*v*_*C*_〉) is coupled (**d**) if certain conditions are satisfied.

In our recent work, we proposed the coupling of waves to the eigenchannels of the *time-gated* reflection matrix as a new type of operator for efficiently focusing waves to a target embedded in a highly scattering medium^25^. Since the previous studies of iterative time reversal operators, or the eigenchannel coupling of a monochromatic transfer matrix, have no temporal gating effect, they were prone to multiple scattering noise. In our previous study, we applied temporal gating in the measurement of the time-gated reflection matrix *R* to reject a large fraction of multiple-scattered waves. We found that this time-gated reflection matrix could be decomposed into two submatrices, i.e. *R* = *T* + *B*. Here, *T* represents a sub-matrix describing multiple-scattered waves having interacted with the target, and *B* represents those having no interaction with the target (Fig. 1a,b). This decomposition provides a new framework since wave propagation is considered a competing process between two multiple scattering processes, *T* and *B*. We demonstrated experimentally and numerically that the coupling of waves to the eigenchannel *R* can induce the preferential coupling of energy to *T* relative to *B* due to the high inter-channel correlation inside *T*.

In the present study, we generalize the competition between two subprocesses and derive an analytic expression describing how the eigenchannel coupling of the total process distributes input energy to individual subprocesses only with the largest eigenvalue and average eigenvalue of each subprocess. For clarity, the eigenchannels |*v*_*X*,*i*_〉 of a given matrix *X* are defined by the column vectors of a unitary matrix *V* obtained from the eigendecomposition of ${X}^{\dagger }X$, i.e. ${X}^{\dagger }X=V\tau {V}^{\dagger }$. Here, *τ* is a square diagonal matrix with non-negative real numbers *τ*_*X*,*i*_ on the diagonal called eigenvalues. By convention, eigenvalues are sorted in the descending order with respect to the eigenchannel index *i*. Therefore, *τ*_*X*,1_ and |*v*_*X*,1_〉 are the largest eigenvalue and its associated eigenchannel, respectively. In our analysis, we consider that two representative parameters of each process, the average eigenvalue 〈*τ*_*X*_〉 and the enhancement factor *η*_*X*_ ≡ *τ*_*X*,1_/〈*τ*_*X*_〉, are known *a priori*. Here, the average eigenvalue 〈*τ*_*X*_〉 corresponds to the average transmittance of the process *X*, and the enhancement factor *η*_*X*_ indicates the effectiveness of the wavefront control in maximizing the transmittance of the corresponding process. We prove that $\chi \equiv \frac{({\eta }_{B}-1)\,\langle {\tau }_{B}\rangle }{({\eta }_{A}-1)\,\langle {\tau }_{A}\rangle }$ is a governing parameter that determines which subprocess is preferable at the time of coupling waves to the eigenchannel of the total process with the largest eigenvalue.

## Principle

In many physical systems, precise knowledge of each subprocess is often difficult to extract because only the total combined process is accessible. The most common example in the field of photonics is the coupling of light to a device composed of multiple devices as depicted in Fig. 1c,d, where two devices are fused at the input and output ends to form a combined device. If individual devices are linear systems, they can be described by the linear transformation operators, *A* and *B*. The merged device is then described by an operator *C* given by

1 $$C=A+B.$$

Here, we investigate the way the first eigenchannel of *C* (|*v*_*C*,1_〉) distributes its energy to the individual sub-channels depending on the properties of *A* and *B*. In particular, we are interested in the way in which the eigenchannels of *A* and *B* determine |*v*_*C*,1_〉.

Let us consider an arbitrary input vector $|{v}_{C}\rangle$. If the input vector was the eigenchannel of the matric *C* with the largest eigenvalue, the total output energy *E*_*C*_ given below would be maximum.

2 $${E}_{C}=\langle {v}_{C}|{C}^{\dagger }C|{v}_{C}\rangle \cong \langle {v}_{C}|{A}^{\dagger }A|{v}_{C}\rangle +\langle {v}_{C}|{B}^{\dagger }B|{v}_{C}\rangle $$

Therefore, we find |*v*_*C*_〉 = |*v*_*C*_〉_*m*_ that maximizes the magnitude of the total output energy *E*_*C*_. Here, the cross terms *A*^†^*B* and *B*^†^*A* are ignored because they are significantly smaller than the other terms when the two matrices are uncorrelated and their average eigenvalues are comparable. In fact, this is quite general in most cases because the two sub-processes are often independent of each other. Moreover, the assumption is valid even until 〈*τ*_*Ai*_〉 ≈ *N*〈*τ*_*Bi*_〉 due to the reduced expectation value of the complex-valued cross terms. On this condition, only the consideration of *A* is good enough since *A* greatly dominates *B* by the factor *N* (see Supplementary Information for details). We will also explore how this operation of maximization affects the energies *E*_*A*_ = 〈*v*_*C*_|*A*^†^*A*|*v*_*C*_〉 and *E*_*B*_ = 〈*v*_*C*_|*B*^†^*B*|*v*_*C*_〉 delivered through individual subprocesses depending on their respective physical properties. If we know the exact transfer matrices *A* and *B*, we can solve the linear algebra problem of the eigendecomposition of *C*^†^*C*, find |*v*_*C*,1_〉, and obtain *E*_*A*_ and *E*_*B*_. However, the exact transfer matrices are not accessible for the case of a fused total process. Nevertheless, we could at least have partial information on the two matrices. For example, we can estimate the average eigenvalues (〈*τ*_*A*_〉 and 〈*τ*_*B*_〉) from the expected signal strength of each process. The enhancement factors (*η*_*A*_ and *η*_*B*_) can also be estimated by the number of effective ‘open’ eigenchannels^26^. The reduction in the effective channel number leads to the increase of *C*^(2)^ correlation, which then induces the increase of the enhancement factor. Thus, we consider that these two representative parameters are known *a priori*, and investigate how they contribute to the determination of |*v*_*C*_〉_*m*_.

To evaluate *E*_*C*_, it is necessary to expand |*v*_*C*_〉 in terms of the eigenchannels of *A* and *B*. On the condition that the average eigenvalues (〈*τ*_*A*_〉 and 〈*τ*_*B*_〉) and the largest eigenvalues (*τ*_*A*,1_ = *η*_*A*_〈*τ*_*A*_〉,*τ*_*B*,1_ = *η*_*B*_〈*τ*_*B*_〉) are given, we make an assumption that eigenvalues other than (*τ*_*A*,1_, *τ*_*B*,1_) have the same magnitude, i.e. ${\tau }_{A,i\ne 1}=\frac{{\sum }_{j=2}^{N}{\tau }_{A,j}}{N-1}=\frac{N\langle {\tau }_{A}\rangle -{\tau }_{A,1}}{N-1}$ and ${\tau }_{B,i\ne 1}=\frac{{\sum }_{j=2}^{N}{\tau }_{B,j}}{N-1}=\frac{N\langle {\tau }_{B}\rangle -{\tau }_{B,1}}{N-1}$. Here, *N* is the number of input channels of *C*. This simplifying assumption considers governing factors for the intuitive understanding of the system, and we discovered that it is valid for the most representative eigenvalue distribution given by the filtered random matrices (FRM)^27^. We can express |*v*_*C*_〉 in terms of the eigenchannels of either *A* or *B* as follows:

3 $$|{v}_{C}\rangle =\sum _{i=1}^{N}\sqrt{{\alpha }_{A,i}}{e}^{i{\varphi }_{A,i}}|{v}_{A,i}\rangle ,$$

4 $$|{v}_{C}\rangle =\sum _{i=1}^{N}\sqrt{{\alpha }_{B,i}}{e}^{i{\varphi }_{B,i}}|{v}_{B,i}\rangle .$$

Here, *α*_*A*,*i*_ and *α*_*B*,*i*_ are set to be real without the loss of generality, and their magnitudes are equal or smaller than unity. Now, let us find *α*_*A*,*i*_, *ϕ*_*A*,*i*_, *α*_*B*,*i*_, and *ϕ*_*B*,*i*_ that maximize *E*_*C*_. After inserting Eqs (3 and 4) into Eq. (2), *E*_*C*_ is expressed as

5 $${E}_{C}={\alpha }_{A,1}{\eta }_{A}\langle {\tau }_{A}\rangle +\frac{(N-{\eta }_{A})\langle {\tau }_{A}\rangle }{N-1}\sum _{i=2}^{N}{\alpha }_{A,i}+{\alpha }_{B,1}{\eta }_{B}\langle {\tau }_{B}\rangle +\frac{(N-{\eta }_{B})\langle {\tau }_{B}\rangle }{N-1}\sum _{i=2}^{N}{\alpha }_{B,i}=\frac{N}{N-1}\langle {\tau }_{A}\rangle [({\eta }_{A}-1){\alpha }_{A,1}-{\eta }_{A}/N+1]+\frac{N}{N-1}\langle {\tau }_{B}\rangle [({\eta }_{B}-1){\alpha }_{B,1}-{\eta }_{B}/N+1].$$

Note that $\sum _{i=2}^{N}{\alpha }_{A,i}=1-{\alpha }_{A,1}$, and any combinations of *α*_*A*,*i*_(i ≠ 1)s result in the same total energy as long as their eigenvalues are the same. As *α*_*A*,1_ and *α*_*B*,1_ increase, *E*_*C*_ also increases because (*η*_*A*_ − 1)〈*τ*_*A*_〉 and (*η*_*B*_ − 1)〈*τ*_*B*_〉 are positive. This concurs with the prediction since *α*_*A*,1_ and *α*_*B*,1_ are the contributions of the largest eigenvalues of *A* and *B*, respectively. However, *α*_*A*,1_ and *α*_*B*,1_ cannot be maximized at the same time because they are not independent of each other. They have a certain relationship due to the condition that |*v*_*C*_〉 in Eqs (3) and (4) should be the same.

If *A* and *B* are two independent operators, their eigenchannels are uncorrelated. In this case, the ensemble average of the squared correlation between eigenchannels of *A* and *B* is 1/*N*: 〈|〈*v*_*A*,*i*_|*v*_*B*,*j*_〉|^2^〉 = 1/*N*. The eigenchannels of *B* can be written as the random superposition of the eigenchannels of *A*:

6 $$|{v}_{B,j}\rangle =\frac{1}{\sqrt{N}}\sum _{i=1}^{N}\sqrt{{c}_{ji}}{e}^{i{\theta }_{ji}}|{v}_{A,i}\rangle ,$$

where $\sqrt{{c}_{ji}}$ accounts for random fluctuations with 〈*c*_*ji*_〉 = 1 and $\sum _{i=1}^{N}{c}_{ij}=N$.

When this equation is inserted into Eq. (4), the coefficients of |*v*_*A*,*i*_〉 need to match those from Eq. (3). By equating the coefficients of |*v*_*A*,1_〉, we can obtain the relationship between *α*_*A*,1_ and *α*_*B*,*i*_ as follows,

7 $${\alpha }_{A,1}=\frac{1}{N}{|\sum _{i=1}^{N}\sqrt{{\alpha }_{B,i}{c}_{i1}}{e}^{i{\varphi }_{B,i}}{e}^{i{\theta }_{i1}}|}^{2}.$$

By inserting Eq. (7) into Eq. (5), *E*_*C*_ can be written as a function of only *α*_*B*,*i*_ and *ϕ*_*B*,*i*_.

Let us now find *α*_*B*,*i*_ and *ϕ*_*B*,*i*_ that maximize *E*_C_. Firstly, the choice of *ϕ*_*B*,*i*_s does not affect *E*_*B*_, the second term of Eq. (5), and it only affects *E*_*A*_, the first term of Eq. (5). Therefore, *ϕ*_*B*,*i*_s maximizing *α*_*A*,1_ will maximize *E*_*A*_, which in turn maximizes *E*_*C*_. *α*_*A*,1_ is maximized when all the phasors in Eq. (7) are aligned; that is, when *ϕ*_*B*,1_ + *θ*_11_ = *ϕ*_*B*,*i*_ + *θ*_*i*1_ for all *i*. This condition yields

8 $${({\alpha }_{A,1})}_{max}=\frac{1}{N}{(\sum _{i=1}^{N}\sqrt{{\alpha }_{B,i}{c}_{i1}})}^{2}.$$

Secondly, the choice of *α*_*B*,*i*_(i ≠ 1)s also does not affect *E*_*B*_ under the assumption that the eigenvalues of *B* other than *τ*_*B*,1_ have the same magnitude, and it only affects *E*_*A*_. Therefore, *α*_*B*,*i*_(i ≠ 1)s maximizing *α*_*A*,1_ will also maximizes *E*_*C*_. By the Cauchy-Schwartz inequality, *α*_*A*,1_ is maximized when $\sqrt{{\alpha }_{Bi}}=\sqrt{\frac{1-{\alpha }_{B,1}}{N-{c}_{11}}}\sqrt{{c}_{i1}}$ for *i* = 2 ~ *N*. This condition yields

9 $${({\alpha }_{A,1})}_{max}=\frac{1}{N}{(\sqrt{{\alpha }_{B,1}{c}_{11}}+\sqrt{N-{c}_{11}}\sqrt{1-{\alpha }_{B,1}})}^{2}.$$

**Figure 2:**
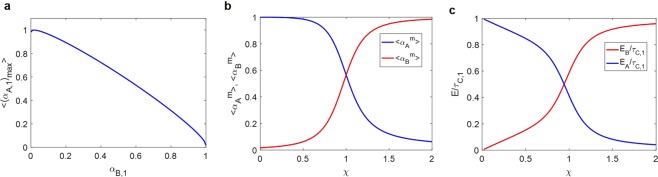
Plots of the obtained analytic equations. (**a**) Maximum of 〈(*α*_*A*,1_)_*max*_〉 for given *α*_*B*,1_ (Eq. (9)) for the case of N = 59. (**b**) $\langle {\alpha }_{A}^{m}\rangle$ and $\langle {\alpha }_{B}^{m}\rangle$ that maximize the total energy (*E*_*C*_) depending on χ (Eqs (11) and (12)). (**c**) The output energy of *A* (*E*_*A*_, blue) and *B* (*E*_*B*_, red) normalized by the total output energy (*E*_*C*_) when |*v*_*C*_〉_*m*_ is coupled.

Therefore, the ensemble average of (*α*_*A*,1_)_*max*_, 〈(*α*_*A*,1_)_*max*_〉, is approximately $\frac{1}{N}{(\sqrt{{\alpha }_{B,1}}+\sqrt{N-1}\sqrt{1-{\alpha }_{B,1}})}^{2}$. This coherent sum of the phasors is maximized when the amplitudes are equally contributed. Thus, (*α*_*A*,1_)_*max*_ is maximum when ${\alpha }_{B,1}=\frac{1\,}{N}$. On the contrary, (*α*_*A*,1_)_*max*_ is minimized when one phasor has a finite amplitude and the other phasors are zero, i.e. *α*_*B*,1_ = 1. Therefore, we can observe the monotonic decrease of 〈(*α*_*A*,1_)_*max*_〉 in the *α*_*B*,1_ range of $\frac{1\,}{N}$ to 1 in Fig. 2a.

Now, *E*_*C*_ is written as a function of only *α*_*B*,1_ by inserting (*α*_*A*,1_)_*max*_ into Eq. (5). Lastly, we can find ${\alpha }_{B,1}={\alpha }_{B}^{m}$ that maximizes *E*_*C*_ by solving $\frac{d{E}_{C}}{d{\alpha }_{B,1}}=0$. At the condition when *N* ≫ 1,

10 $${\alpha }_{B}^{m}\approx \frac{1}{2}+\frac{1}{2}\frac{\chi -1}{\sqrt{\frac{4{c}_{11}}{N}+{(\chi -1)}^{2}}}.$$

The ensemble averaged ${\alpha }_{B}^{m}$ can be approximated as

11 $$\langle {\alpha }_{B}^{m}\rangle \approx \frac{1}{2}+\frac{1}{2}\frac{\chi -1}{\sqrt{\frac{4}{N}+{(\chi -1)}^{2}}}.$$

By inserting Eqs (10) into (9), we can also obtain the ensemble average of ${\alpha }_{A,1}={\alpha }_{A}^{{\rm{m}}}$ which maximizes *E*_*C*_:

12 $$\langle {\alpha }_{A}^{m}\rangle \approx \frac{1}{2}+\frac{1}{2}\frac{\frac{1}{\chi }-1}{\sqrt{\frac{4}{N}+{(\frac{1}{\chi }-1)}^{2}}}\,.$$

Here, we define $\chi \equiv \frac{({\eta }_{B}-1)\,\langle {\tau }_{B}\rangle }{({\eta }_{A}-1)\,\langle {\tau }_{A}\rangle }\,$, which is a critical parameter determining which subprocess would be more beneficial for maximizing the total energy. The numerator of *χ* refers to the increment in energy transmission relative to the average transmittance when |*v*_*B*,1_〉 is coupled. Its denominator has the same physical meaning when |*v*_*A*,1_〉 is coupled. Therefore, when χ < 1, |*v*_*A*,1_〉 is more likely to be coupled such that $\langle {\alpha }_{A}^{m}\rangle$ becomes larger than $\langle {\alpha }_{B}^{m}\rangle$. When χ > 1, the opposite is the case. In Fig. 2b, $\langle {\alpha }_{A}^{m}\rangle$ and $\langle {\alpha }_{B}^{m}\rangle$ are plotted as a function of *χ*, where we can observe their crossover at *χ* = 1. As expected, $\langle {\alpha }_{B}^{m}\rangle$ converges to unity and $\langle {\alpha }_{A}^{m}\rangle$ to zero as *χ* is increased, and vice versa if *χ* is reduced.

Using $\langle {\alpha }_{A}^{m}\rangle$ and $\langle {\alpha }_{B}^{m}\rangle$, we can write down the largest eigenvalue of *C* in terms of the average eigenvalues and the enhancement factors of *A* and *B*,

13 $${\tau }_{C,1}\approx [({\eta }_{A}-1)\langle {\alpha }_{A}^{m}\rangle +1]{\tau }_{A}+[({\eta }_{B}-1)\langle {\alpha }_{B}^{m}\rangle +1]{\tau }_{B}.$$

Since $\langle {\alpha }_{A}^{m}\rangle$ becomes larger than $\langle {\alpha }_{B}^{m}\rangle$ when *χ* < 1, *E*_*A*_ becomes larger than *E*_*B*_. The contribution of *E*_*A*_ and *E*_*B*_ to *τ*_*C*,1_ is plotted in Fig. 2c. When *χ* = 1, that is when (*η*_*A*_ − 1)〈*τ*_*A*_〉 = (*η*_*B*_ − 1)〈*τ*_*B*_〉, the energy is almost equally distributed to *A* and *B*.

## Validation of analytic solution by comparing it with numerical results

**Figure 3:**
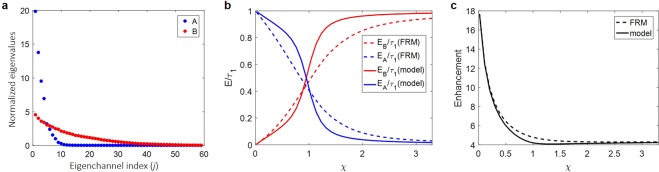
Validation with filtered random matrix ensemble. (**a**) Normalized eigenvalues *τ*_*A*,*i*_/〈*τ*_*A*_〉 (blue dots) and *τ*_*B*,*i*_/〈*τ*_*B*_〉 (red dots) of *A* and *B*, respectively. (**b**) Output energy of *A* (*E*_*A*_, blue) and *B* (*E*_*B*_, red) normalized by the total output energy (*E*_*C*_) when |*v*_*C*,1_〉 is coupled. Dashed lines are from the numerical simulation, and solid lines are from the analytic function. (**c**) Dashed and solid curves indicate the energy enhancement (*η*_*C*_ ≡ *τ*_*C*,1_/〈*τ*_*C*_〉) of matrix *C* calculated from numerical simulation and analytic function, respectively.

To validate the ability of the derived analytic function, |*v*_*C*_〉_*m*_, in predicting the exact solution |*v*_*C*,1_〉, we considered the FRM as exemplary matrices of *A* and *B*. Most of experimentally measurable matrices can be regarded as the FRM ensemble having the limited channel control and the correlation induced by multiple scattering. We generated 100 sets of the full 500 × 500 random matrices, and selected 200 output and 59 input channels; we thus obtained a 200 × 59 FRM ensemble. Here, *A* and *B* have two different arbitrary eigenvalue distributions (ensemble averaged) as shown in Fig. 3a, where their eigenchannels are set as uncorrelated to satisfy the assumption in Eq. (6).

In order to validate the analytic function for various *χ*s, we constructed various *C*(*α*) matrices by adjusting the relative average eigenvalue of matrix *A*, i.e. *C*(*α*) = *αA* + *B*. First, we found *τ*_*C*,1_ and |*v*_*C*,1_〉 from the singular value decomposition of *C*(*α*) using the Matlab built-in function. We then obtained ${E}_{A}=\langle {v}_{C,1}|{A}^{\dagger }A|{v}_{C,1}\rangle ,\,{E}_{B}=\langle {v}_{C,1}|{B}^{\dagger }B|{v}_{C,1}\rangle ,$ and the total enhancement (*η*_*C*_ ≡ *τ*_*C*,1_/〈*τ*_*C*_〉) of *C*(*α*) for each set of the FRM ensemble. We then compared the ensemble averaged results with those from our analytic function. The contribution of *E*_*A*_ and *E*_*B*_ to *τ*_*C*,1_is plotted in Fig. 3b. Dashed lines are from the numerical simulation, and solid lines are from the analytic solution. We observed reasonable agreements between the analytic solution and numerical simulation. In other words, we can closely estimate the energy distribution of |*v*_*C*,1_〉 to each subprocess with the analytic solution derived using only partial information of the subprocesses. The slight discrepancy is due to the assumption that eigenvalues other than (*τ*_*A*,1_, *τ*_*B*,1_) have the same magnitude. We also compared the energy enhancement of the total process *C* (*η*_*C*_ ≡ *τ*_*C*,1_/〈*τ*_*C*_〉) obtained from the numerical simulation solution and the analytic function (Fig. 3c), and found good agreement between them throughout the wide range of *χ*. This demonstrates that the analytic solution |*v*_*C*_〉_*m*_ we derived closely predicts the eigenchannel |*v*_*C*,1_〉.

### Validation of the analytic function for the physical systems

To date, we have verified that the derived analytic function reasonably predicts the exact solution for |*v*_*C*,1_〉 of arbitrary matrices. Here, we verify the ability of the analytic function in predicting the exact solution for |*v*_*C*,1_〉 of matrices from a real physical system. We consider the time-gated reflection matrix (*R*) of light pulse coupled to a highly scattering medium with an embedded target^25^, as presented in the introduction. This matrix can be expressed as a summation of two submatrices, a submatrix of the waves having interacted with the target (*T*), and the other submatrix of the waves having no interaction with the target (*B*).

**Figure 4:**
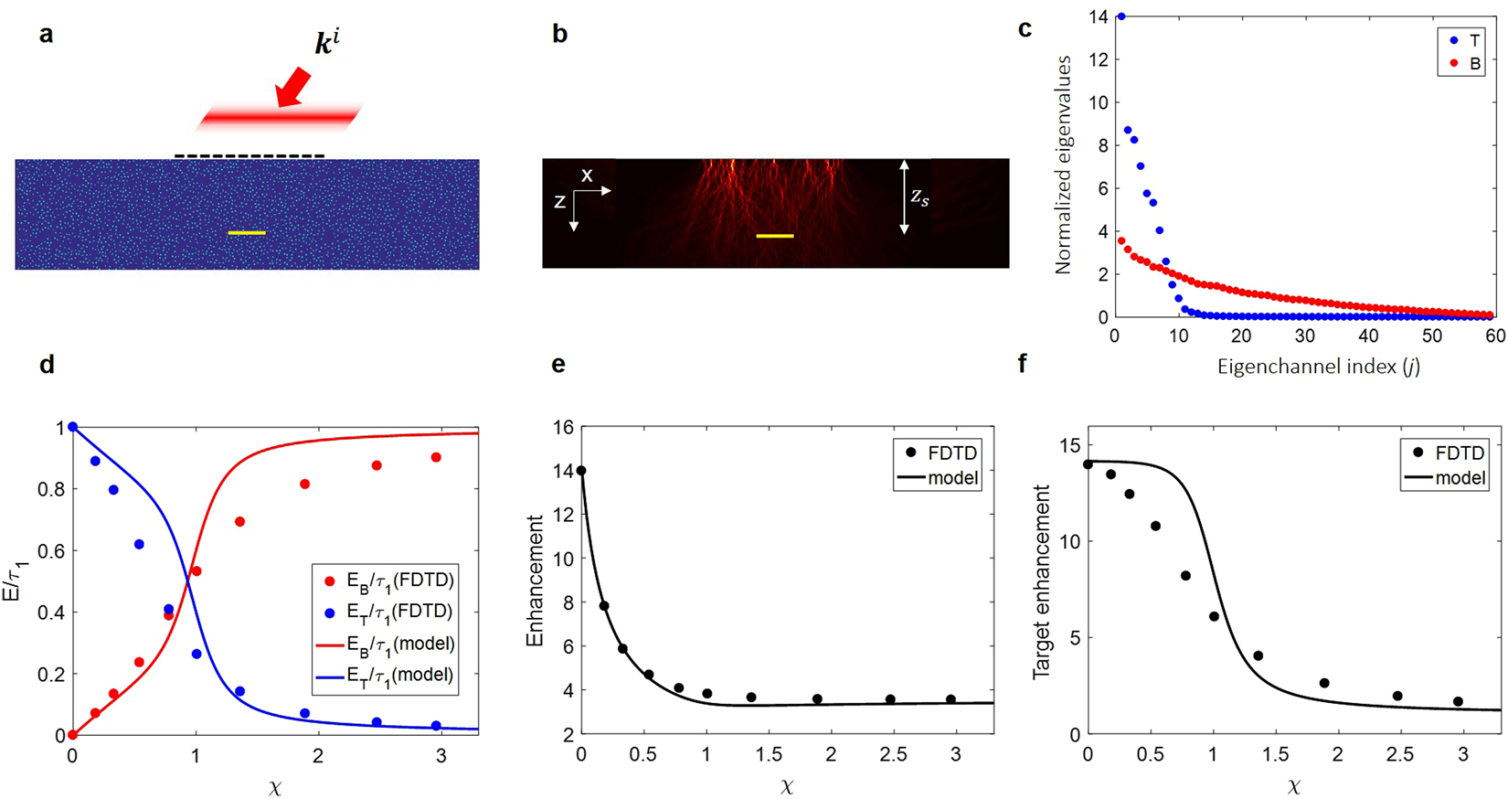
Validation with finite-difference time-domain (FDTD) simulations. (**a**) Schematic configuration of FDTD simulation. Pulsed wave with a planar wavefront illuminates the center of the scattering medium from the top. The transverse wavevector of the incident light is indicated as ${{\bf{k}}}^{i}$. Note that the temporal pulse front, of which the width is 6.7 fs, is set parallel to the *x* axis regardless of ${{\bf{k}}}^{i}$. Black dashed line: 40 μm-width window through which an incident wave was illuminated and backscattered waves were measured. Target (yellow line) size, 10 μm. (**b**) Accumulated intensity map of the forward propagating waves for a random input. (**c**) Normalized eigenvalues *τ*_*T*,*i*_/〈*τ*_*T*_〉 (blue dots) and *τ*_*B*,*i*_/〈*τ*_*B*_〉 (red dots) of *T* and *B*, respectively. (**d**) Output energy of *T* (*E*_*T*_, blue) and *B* (*E*_*B*_, red) normalized by the total output energy (*E*_*R*_) when |*v*_*R*,1_〉 is coupled. Circles are from FDTD simulation, and lines are from the analytic function. (**e**) Enhancement (*η*_*R*_ ≡ *τ*_*R*,1_/〈*τ*_*R*_〉) of total reflection matrix *R* calculated from FDTD simulation (circles) and the analytic function (solid curve). (**f**) Enhancement of energy delivery to the target when waves are coupled to |*v*_*R*,1_〉 for the cases of FDTD simulation (circles) and the analytic function (solid curve).

We performed numerical simulations of wave propagation using the finite-difference time-domain (FDTD) method. We numerically prepared a target object with a 10 μm width and various reflectances from 3.5% to 60%. We then placed the target at a depth of z_s_ = 21 μm from the surface of the scattering medium, the scattering and transport mean free paths of which are *l*_*s*_ = 5.1 μm and *l*_*t*_ = 20.8 μm, respectively. Light pulses with the pulse width of 6.7 fs were sent through a 40 μm-width window in the middle of the scattering medium (dashed black line in Fig. 4a) and the backscattered waves were detected through the same window. For each incident wave vector, ${{\bf{k}}}^{i}$, we computed the wave propagation and recorded the reflected wave as a function of flight time. A time-gated reflection matrix was constructed from the recorded maps.

Even though we cannot distinguish *T* and *B* in a real experiment, we can systematically separate out *T* and *B* from *R* in FDTD simulations. We calculated *B* in the absence of a target object and acquired *T* by the relation, *T* = *R* − *B*. We found eigenvalues of *T* and *B*, *τ*_*T*,*i*_ and *τ*_*B*,*i*_ (Fig. 4c). We observed that the enhancement factor of *T* (*η*_*T*_)is larger than that of *B* (*η*_*B*_). As reported earlier, the number of effective channels in *T* is reduced because the target multiple-scattered waves should be reflected by a small target area on their return to the detector, while there is no such constraint for *B*^25^.

In order to validate the analytic function for various χ’s, we analyzed *R *from the FDTD simulations using the same method as that for the analysis performed for the FRM matrices. The contribution of *E*_*T*_ and *E*_*B*_ to *τ*_*R*,1_ (Fig. 4d) and the total enhancement (Fig. 4e) were obtained from the FDTD simulations (circles) and compared to the analytic functions (solid curves). From these comparisons, we conclude that the analytic function also closely predicts the exact solution of the matrices derived from a physical system.

In this particular example, we are interested in efficiently focusing waves onto the target embedded in a highly scattering medium. This focusing can be optimized if we find and couple the first eigenchannel of *T*, |*v*_*T*,1_〉, since it will maximize the reflection intensity from the target. However, it is not possible to find |*v*_*T*,1_〉 because the reflection from the target (*T*) cannot be separated from the reflection from the background (*B*). Thus, we presented that coupling the first eigenchannel of the total matrix (|*v*_*R*,1_〉) can enhance the energy delivery to the target if certain conditions are satisfied. In the previous study, this condition was predicted only from experimental and numerical observations. Using the derived analytic function, we can now analytically derive the working condition of the previously proposed method.

To quantify the degree to which the coupling of the wave to |*v*_*R*,1_〉 enhances the energy delivery to the target, we define the target enhancement factor, *η*, which is the output intensity of *T* matrix when the input is |*v*_*R*,1_〉 with respect to the average output intensity of *T*. At the condition when *N* ≫ 1,

14 $$\eta \equiv \frac{\langle {v}_{R,1}|{T}^{\dagger }T|{v}_{R,1}\rangle }{\langle {\tau }_{T}\rangle }\approx {\alpha }_{T,1}({\eta }_{T}-1)+1.$$

In Fig. 4f, this target enhancement is shown from the FDTD simulations (circles) and from the analytic functions (solid curve). This curve visualizes the working condition of the suggested method. The target enhancement rapidly decreases at around *χ* = 1. From this analytic equation, we can now suggest *χ* = 1 as the working condition of the previously proposed method, where

15 $$\eta \approx \frac{{\eta }_{T}+1}{2}.$$

The target enhancement by |*v*_*R*,1_〉 becomes approximately half of the maximum enhancement achievable by the first eigenchannel of *T*. In our FDTD simulations, *η*_*T*_ and *η*_*B*_ are 13.98 and 3.54, respectively. Therefore, *η* is still about 7.5, even when 〈*τ*^*B*^〉 is larger than 5 times 〈*τ*^*T*^〉, at which *χ* = 1.

One of the critical assumptions we made in our derivation was that eigenvalues other than the largest have the same magnitude, i.e. ${\tau }_{A,i\ne 1}=\frac{{\sum }_{j=2}^{N}{\tau }_{A,j}}{N-1}=\frac{N\langle {\tau }_{A}\rangle -{\tau }_{A,1}}{N-1}$ and ${\tau }_{B,i\ne 1}=\frac{{\sum }_{j=2}^{N}{\tau }_{B,j}}{N-1}=\frac{N\langle {\tau }_{B}\rangle -{\tau }_{B,1}}{N-1}$. While this assumption was inevitable since only partial information is known *a priori*, it serves as the main source of discrepancy between the analytic equation and exact solution. We checked the validity of this assumption by considering three different eigenvalue distributions of matrix *B*, of which the widths are progressively increased with respect to the eigenchannels index. For simplicity, *η*_*B*_, 〈*τ*_*B*_〉, and the eigenvalue distribution of *A* were fixed. We then estimated the discrepancy between the eigenvalue distribution of *B* and that of our assumption by the average of the absolute difference between the two distributions normalized by the average eigenvalue. For the three different matrices of *B*, the discrepancies were 85%, 111%, and 148%. The corresponding errors in target enhancement *η* of *A* when 0 ≤ *χ* ≤ 1 were 6.7%, 10.0%, and 14.7%. As expected, an error in *η* depends on the error in distribution. Even though the error in distribution is very large, the error in *η* is relatively small. We performed additional analysis with FRM. We fixed the eigenvalue distribution of A matrix with *η*_*A*_ = 17.2 and constructed B matrices with various enhancement factors ranging from *η*_*B*_ = 2.5 to *η*_*B*_ = 14.5. The errors in target enhancement were less than 10% for this wide range of eigenvalue distributions. These tests demonstrate that the assumption we made in our theoretical derivation is quite robust and our model can be useful even when an eigenvalue distribution deviates from our assumption.

Even when eigenvalues other than the largest are not the same, the choice of *ϕ*_*B*,*i*_s in Eq. (7) does not affect *E*_*B*_, and finding *ϕ*_*B*,*i*_s maximizing *α*_*A*,1_ results in phase matching between |*v*_*C*_〉 and |*v*_*A*,1_〉, which already provides a large overlap between |*v*_*C*_〉 and |*v*_*A*,1_〉 before tuning *α*_*B*,*i*_s. Since this phase matching is the leading order in maximizing *E*_*C*_, our simple model remains largely valid. This logic is in line with the iterative phase conjugation process for finding the first eigenvector, where the first eigenvalues are the governing factors as the iteration weights the first eigenvectors. Still, finding the detailed solution for *α*_*B*,*i*_s when eigenvalues other than the largest are not the same could be an interesting future study.

## Conclusion

We presented a new analytic framework for understanding the coupling of waves to a total process *C* composed of two competing subprocesses, *A* and *B*. The derived analytic equation explains how the eigenchannel coupling of the total process distributes input energy to each subprocess on the condition that only partial information on each subprocess such as the average eigenvalue (〈*τ*_*A*_〉, 〈*τ*_*B*_〉) and enhancement factor (*η*_*A*_, *η*_*B*_) are known. We found that $\chi \equiv \frac{({\eta }_{B}-1)\,\langle {\tau }_{B}\rangle }{({\eta }_{A}-1)\langle \,{\tau }_{A}\rangle }$ is a governing parameter that determines which subprocess is preferable at the time of coupling waves to the eigenchannel of *C* with the largest eigenvalue. Input energy is preferably coupled to process *A* if *χ* is smaller than unity, and process B if *χ* is larger than unity. In many cases, the partial information can be estimated even when the exact transfer matrix of each process is not accessible. Therefore, our analysis allows us to predict the distribution of wave energy to two inseparable subprocesses. The validity of the derived equation was supported by the exact solutions of the transfer matrices from the FDTD simulations as well as the FRM matrices. The framework is so general that it can be applied to many types of competing wave propagations, provided their interaction is linear.

## Supplementary information

Supplementary Information
