# Supplementary material for: Analytic framework for understanding the competing multiple light scattering processes
Source: Sci Rep. 2019 Feb 26;9:2785. doi: 10.1038/s41598-019-39165-7 (PMC6391453; doi:10.1038/s41598-019-39165-7)
Supplement: Supplementary file 1 — Supplementary Information [file 41598_2019_39165_MOESM1_ESM.pdf]

Analytic framework for understanding the competing multiple light scattering processes

Ye-Ryoung Lee[1,2], Wonjun Choi[1,2], Seungwon Jeong[1,2], and Wonshik Choi[1,2,*]

[1]*Center for Molecular Spectroscopy and Dynamics, Institute for Basic Science, Seoul 02841, Korea*
[2]*Department of Physics, Korea University, Seoul 02841, Korea*

Cross terms in Eq. (2)

Here, we show that the cross terms in Eq. (2) are negligible compared to the other terms. For the comparison between the cross terms and self-interference terms, we express each term in Eq. (2) in terms of $\langle\tau_A\rangle$ and $\langle\tau_B\rangle$ in the following.

Let us define the coefficients of Eqs. (3) and (4) as $c_{Ai} = \sqrt{\alpha_{A,i}}e^{i\phi_{A,i}}$, $c_{Bi} = \sqrt{\alpha_{B,i}}e^{i\phi_{B,i}}$. Then, the cross terms can be written as

$$\langle v_c|A^+B|v_c\rangle + \langle v_c|B^+A|v_c\rangle = \langle v_c|A^+B|v_c\rangle + \langle v_c|A^+B|v_c\rangle^+$$

$$= 2\,Re\left(\sum_{i=1}^{N}\sum_{j=1}^{N} c_{Ai}^{*}\sigma_{Ai}c_{Bj}\sigma_{Bj}\langle u_{Ai}|u_{Bj}\rangle\right). \quad (S1)$$

Here σ_{Ai} and σ_{Bi} are the singular values, and $|u_{Ai}\rangle$ and $|u_{Bi}\rangle$ are the output eigenvectors corresponding to the input eigenvectors, $|v_{Ai}\rangle$ and $|v_{Bi}\rangle$, respectively. When A and B are two independent operators, the output eigenvectors of B can be written as the random superposition of the eigenchannels of A:

$$|u_{Bj}\rangle = \frac{1}{\sqrt{N}}\sum_{i=1}^{N}\sqrt{d_{ji}}e^{i\phi_{ji}}\,|u_{Ai}\rangle, \quad (S2)$$

where $\sqrt{d_{ji}}$ accounts for random fluctuations with $\langle d_{ji}\rangle = 1$ and $\sum_{i=1}^{N} d_{ij} = N$.

Then,

$$\langle v|A^{+}B|v\rangle + \langle v|B^{+}A|v\rangle = 2\,Re\left(\sum_{i=1}^{N}\sum_{j=1}^{N} c_{Ai}^{*}\sigma_{Ai}c_{Bj}\sigma_{Bj}\frac{1}{\sqrt{N}}\sqrt{d_{ji}}e^{i\phi_{ji}}\right) \quad (S3)$$

The remained terms in Eq. (2) expand as follows

$$\langle v|A^{+}A|v\rangle + \langle v|B^{+}B|v\rangle = \sum_{i=1}^{N}|c_{Ai}\sigma_{Ai}|^{2} + \sum_{i=1}^{N}|c_{Bi}\sigma_{Bi}|^{2} \quad (S4)$$

In order to compare the magnitudes of Eq. (S3) and Eq. (S4), let us approximately express each term in terms of the average eigenvalue, $\langle\tau_{Ai}\rangle$ and $\langle\tau_{Bi}\rangle$. $\langle v|A^{+}A|v\rangle$ and $\langle v|B^{+}B|v\rangle$ in Eq. (S4) are on the order of $\langle\tau_{Ai}\rangle$ and $\langle\tau_{Bi}\rangle$, respectively. When $c_{Ai}^{*}c_{Bj}e^{i\phi_{ji}}$s have random phases, the amplitude of the term inside the parenthesis in Eq. (S3) is on the order of $\sqrt{\langle\tau_{Ai}\rangle}\sqrt{\langle\tau_{Bi}\rangle}/\sqrt{N}$. In this case, the maximum value of the cross terms is on the order of $2\sqrt{\langle\tau_{Ai}\rangle}\sqrt{\langle\tau_{Bi}\rangle}/\sqrt{N}$. When $\langle\tau_{Ai}\rangle$ and $\langle\tau_{Bi}\rangle$ are comparable, the cross terms are smaller than Eq. (S4) by a factor of $\sqrt{N}$. Thus, the cross terms are negligible when N is large enough. If $\langle\tau_{Bi}\rangle$ is much smaller than $\langle\tau_{Ai}\rangle$, the maximum value of the cross terms become comparable to the $\langle v|B^{+}B|v\rangle$ in Eq. (S4) only when $\langle\tau_{Ai}\rangle/\langle\tau_{Bi}\rangle > N/4$. On this condition, both the cross terms and $\langle v|B^{+}B|v\rangle$ in Eq. (S4) become negligible compared to $\langle v|A^{+}A|v\rangle$ in Eq. (S4). Therefore, we don't need to consider the competition between A and B anymore since A plays a dominant role in determining the eigenchannels of C.
